# A Cultural Memory of the Digital Age?

**DOI:** 10.1007/s11196-020-09778-7

**Published:** 2020-10-06

**Authors:** Dario Henri Haux, Antoinette Maget Dominicé, Jana Alexandra Raspotnig

**Affiliations:** 1 University of Lucerne, Lucerne, Switzerland; 2 LMU Munich, Munich, Germany

**Keywords:** Digital cultural heritage, Cultural memory, Digital practices, Law & humanities

## Abstract

Considering digital cultural heritage as the digitalized assets from memory institutions and digital born art, this paper aims to build on its current normative definitions. This first notion addresses the subtle, yet complex relationship between technology and culture. In addition, we consider the criteria set for defining heritage in memory theorization. By doing so, we want to challenge the lack of uniform standards and approaches in dealing with digital cultural heritage and to give Aleida and Jan Assmann's Theory of Cultural Memory a normative dimension. Can there be a cultural memory of the digital age?

## Introduction

Our everyday life is shaped by digital technologies. Becoming unavoidable, we encounter them in all contexts. In the world of culture, the rises of digital initiatives within museums, galleries, libraries and other memory institutions allow to (partially) visit museums and/or temporary exhibitions, to access heritage resources. In some way, some of these heritage institutions furthermore open up the possibility of reusing parts of the works. Digitized cultural assets kept in public and private collections can be downloaded and edited, while artists are creating art from digital devices or integrate tech gadgets and images in installations [1]. By the beginning of 2020, with the rise of COVID-19, these developments have become a new impulse, as closed heritage institutions had to re-invent their interaction with the public and users who were constrained to stay at home. In this way, tangible resources and “localized” experiences gained a further dimension. Nonetheless, this impulse relies on many unanswered questions. The exponential digitization of contents, resources and presentations, as well as the creation of digital art raised concerns on the definition, the preservation and the transmission of this heritage. At the same time, however, these concerns are mainly of conceptual nature, as the digital cultural heritage is still in a need of a narrower definition. They are also of structural, more material nature [2] and finally challenge neocolonialist [3, 4] and monopolistic [5] positions.

What may be understood as digital cultural heritage builds part II of this article and aims to set the line between heritage and social practices. By referring to a non-exhaustive presentation of some exemplary cases in heritage institutions and the art world more in general, we further define the topic of our paper. Here, we distinguish traditional forms, participative cultural practices and creations on social media. Even if most of these latter endeavors are closely linked to the traditional notion of culture, to a great extent they can be classified as mass culture [6]. In part III we refer to existing legal approaches to digital cultural heritage and the UNESCO Charter of 2003. Part IV then refers to general criteria set out to define cultural memory and its constitutive elements and handlings: starting from a non-locative intangible perception of a monument as introduced by Pierre Nora, we dive deeper into the (digital) interpretation by memory theorists like Jan and Aleida Assmann, for then identifying their normativity. Finally, in part V and building up on norms of law and normative of memory, we look at the relevant ‘contact zones’ [7, p. 188] in and around digital cultural heritage.

## Cultural Heritage in and From the Digital Age

Considerations on cultural heritage, memory institutions and digitization have been relevant for a long time, begin discussed during the first growth of the Internet [8] and embracing many disciplines [9, 10]. Some points are worthy to be highlighted, as these processes underwent many transformations.

Digitization has been integrated in memory institutions as a way to contribute to the preservation of cultural objects, to their documentation in form of a digitization of their appearance and all archives related to, and finally to enhance the access to. Currently, in a world dominated by digital initiatives, heritage institutions go various digital ways, depending on their collections, their locations, and their resources [11]. There are many differences, varying from country to country [12, p. 1122].

One can identify ‘four concepts central in developing digital strategies for heritage collections: digitization, metadata indices, intellectual property rights management and business models [13, p. 157]. This gained a further dimension with the development of the virtual and new presentations solutions. In exhibitions and educational practices, traditional practices of the analogue world encounter the digital. This way, tangible works converted by scientists, artists, institutions, and other actors become intangible—and remain tangible at the same time [14, p. 39]. The resulting variations make it possible to work on the digital form without causing any damage to the original. It is possible to switch back and forth between old and new. This way, creativity and experimental ambition is further triggered and encouraged. Hence, it becomes clear that there are numerous advantages to digitization. Not only has the number of museum visitors been rising for years [15], but many are also inclined to take part in this digital cultural venture [16, 17]. The interest in old and new forms of culture seems to be growing steadily and appealing to more and more individuals from different backgrounds.

These three aspects of preservation, documentation, and access may get bundled in some specific cases, such as the resolution of the litigation concerning the Bibliotheca Palatina. This approach is highly dominated by conservation concerns, as „Conservation is […] essential in defining and maintaining cultural value […]. Conservation can serve to reveal hidden information, it can increase the lifetime of an object, and enable increased enjoyment and understanding of cultural material. Conservation of cultural material is an intrinsic part of our need as a society to collect, organize and display culture. It is a complex intellectual and physical process that raises many ethical, technical and philosophical challenges; and it is through those challenges that we gain a better understanding of the world as it was, as it is, and to some degree, will be.“ [18, p. 174–175].

The increasing possibilities opened by digital media also infiltrate the art scene, contributing to the development of new works of art [19]. These challenge the traditional structures of conservation, storage and presentation, as their relation to materiality and time are threatened a.o. by obsolescence [20]. Besides such artistic practices, digital testimonies and practices gain in importance for memory institutions. A particularly significant example is the archiving of all Tweets by the Library of Congress [21] and much narrower, of digital objects and digital solutions in museums produced during the lockdowns due to the covid-19 pandemic all around the world [22–24]. Not just digitization of artworks, but also contemporary collecting projects, social media and streaming initiatives, virtual tours and online exhibitions have recently shaped the digital artworld [23]. Particularly history museums and city museums called the population for testimonies, to be sent for integration in the standing collections. By doing so, institutions are contributing to define a new category of heritage, a purely communicational heritage [25, p. 260]. And by collecting material, these institutions recognized the significance of all forms of expression during lockdowns and contribute to make some of the material available to the public in the future [26, p. 2]. That is why the process of digitization is often described as fundamentally democratic: everything is and will be open and accessible for everyone [27, p. 4]. This western centered position has to be dampened, in a time where still a large part of the global population cannot access the internet and where the rest of the internet users are facing different barriers such as paywalls. At the same time, this integration of digital cultural items may be understood as a counterpart to the general question of who owns the internet [5], which would go far beyond the scope of this article.

The integration of digital practices within memory institutions and artistic practices may be differently understood and “be explored through multiple optics” [28, p. 66]. When considering digital art works [29] and digital items, they have to be differentiated from the so-called digitization, as these—“reproductions” of cultural objects and documentations, as well as digital access of art projects [30, 31]—will never be compared to analog components. All other digital cultural items have a material pendant but are much more than copy challenging our notion of authenticity, they are new things [32]. And as such, they got the attention of the UNESCO Member States, as they got recognized as being a facet of our cultural heritage but still undefined and facing new threats.

## International Normative Attempt and the UNESCO Charter on the Preservation of Digital Heritage

As aforesaid, the delineation of “a” digital heritage is challenged by digital technologies. By shaping new dimensions of cultural heritage and adding emerging categories of normative thinking, the recognition of practices and in this way, the classification within the current legal system, is further blurred. To name an example, in intellectual property law and especially within its reference to the cultural practice of music sampling, the boundaries between inspiration and intellectual *theft* become increasingly opaque [33]. The same applies to social media posts, that as to date, have not been undergone thorough legal classification. In this context and in the light of the seismic development of the digital, both as a mean and as a category, it seems central to find a normative definition for digital heritage.

Building on programs aiming to preserve information and documents as a support of memory, UNESCO gave a first decisive impulse at the twist of the twenty-first century. During its 32nd session, the general conference of UNESCO on 17 October 2003 adopted the so-called Charter on the Preservation of Digital Heritage. The adoption and its reception attest of many crossovers with the Memory of the World program and its implementation [34, p. 164]. The 2003 Charter follows a four-part structure, further subdivided in twelve articles, preceded by a short preamble. Whilst the first part offers a definition of digital heritage and its access, the second part underlines the corresponding threats. Possible measures and responsibilities of the different stakeholders, including UNESCO, constitute Part III and IV of the Charter respectively.

As one of the first international normative texts in this field, Sect. 1 of the Charter attempts to provide a definition of digital heritage, building up on a standard, broadly set classification and overtopped by the idea of a common heritage. Art. 1 para 1 states that:

“The digital heritage consists of unique resources of human knowledge and expression. It embraces cultural, educational, scientific and administrative resources, as well as technical, legal, medical and other kinds of information created digitally, or converted into digital form from existing analogue resources. Where resources are ‘born digital’, there is no other format but the digital object”.

According to this definition, digital heritage may derive from various sources and practices. At the same time, no qualitative criteria have to be met. This can be seen as a major shift, especially when compared to other UNESCO instruments. Furthermore, the definition incorporates the dual nature of objects linked to an analogue resource and at the same time being self-reliant, but fails to refer to material aspects, such as the hardware needed. However, these material components are evoked in Art. 3 Para 2. Within Art. 1 para. 2 the formats are listed, including their still unknown diversities:

“Digital materials include texts, databases, still and moving images, audio, graphics, software and web pages, among a wide and growing range of formats. They are frequently ephemeral, and require purposeful production, maintenance and management to be retained”.

The necessary support for its sustainability closes Art. 1 para. 2 and recalls the elusive nature of digital heritage. The combination of different formats and transient nature is one of many challenges, as further stated in Art. 1 para. 3, which call on the importance of digital heritage for the human mankind and on its easiest, fullest and widest access:

“Many of these resources have lasting value and significance, and therefore constitute a heritage that should be protected and preserved for current and future generations. This ever-growing heritage may exist in any language, in any part of the world, and in any area of human knowledge or expression”.

This introductory article of the Charter can be criticized as being an all-encompassing attempt, but yet too indefinite. On the other hand, it avoids the trap of an itemization of a heritage under which is under permanent development. Besides that, it is positive to note that this approach attempts to base the model on a very broad foundation that includes a large number of works. In this way, the Charter aims to be all-encompassing. At the same time, however, it is also clear that a broad definition will not lead to greater recognition of the digital as a heritage to be equally considered as the world heritage sites or the intangible cultural heritage. The inclusion of the digital in the current heritage programs appears to be limited to the documentation of other heritage objects, as recommended in Chap. IV Sect. 1.3 Para 119 of the Operational Guidelines for the Implementation of the Convention for the Safeguarding of the Intangible Cultural Heritage [35]. The development of further UNESCO Programs merges some of the aspects of preservation of a digital heritage, even if mostly offering partial overlapping: if the Charter is closely linked with the action of the UNESCO to safeguard the documentary heritage [36, p. 38], the effective relationship with other programs remain unclear [37, p. 160]. These programs are the UNESCO Memory of the World Program (MoW), the Platform to Enhance the Sustainability of the Information Society Transglobally (PERSIST) launched 2013 by the UNESCO and the Recommendation concerning the preservation of, and access to, documentary heritage including in digital form adopted in 2015 [38], as well as the recently launched Software Heritage Project, jointly lead by UNESCO and the French National Research Institute for the Digital Sciences (INRIA) [39].

In terms of governance, for example, one may identify some similarities between these initiatives and the Charter. MoW, Persist and the Software Heritage Project all are designed as experts-guided programs, instead of being mostly determined by state parties. The role of stakeholders and their implication in the preservation of the digital heritage are underlined in the Charter, with a strong accent on cooperation in all aspects of selection, preservation and access to digital heritage. This is a tribute to the strong involvement of the memory institutions in this field, like libraries, far before being a topic on the international political agenda. At the same time and through the open qualification of digital heritage—freed of the outstanding criteria known for other heritage objects in further UNESCO fields of action—the Charter is in line with the MoW program. The later has been launched 1992 by the UNESCO and aims to contribute to the safeguard of documentary heritage, to ensure access to and to increase awareness of the existence and significance of documentary heritage. As stated by Folarin Shyllon 2012, after the Expert meeting on the 20th anniversary of UNESCO’s MoW, ‘the absence of a normative instrument to pilot the MoW may account for the lack of the visibility of it outside UNESCO in contrast to the other two heritage programs, namely, the World Heritage Convention and Intangible Heritage Convention’ [40, p. 576]. A similar analysis may be done for the digital heritage: Almost twenty years after its adopting, the Charter hasn’t been further developed and many aspects diluted in other programs, like the Software Heritage program.

For coming back to the Charter on the Preservation of the Digital Heritage, it becomes clear that the text challenges an established conception of the digital. The shift away from a purely archival and informational orientation [28, p. 55], towards a combination of static archiving and dynamic forms of memory is hereby central. By doing so, the UNESCO is trying to avoid mandating a retrospective-archival strategy but prefers one that is primarily future-oriented. This is reflected in the reference to the hardware and the software, as stated in Art. 3 concerning the threat of loss:

“The world’s digital heritage is at risk of being lost to posterity. Contributing factors include the rapid obsolescence of the hardware and software which brings it to life, uncertainties about resources, responsibility and methods for maintenance and preservation, and the lack of supportive legislation”.

“Attitudinal change has fallen behind technological change. Digital evolution has been too rapid and costly for governments and institutions to develop timely and informed preservation strategies. The threat to the economic, social, intellectual and cultural potential of the heritage—the building blocks of the future—has not been fully grasped”.

Whilst the second passage is referring to general goals of the Charter, the first part precisely points out that when safeguarding the digital heritage, not only the content, but also the technical devices play a crucial role. Here, a reference to concepts of "interoperability" [41] can be made, by means of which attempts are made to secure points of contact for the underlying technology. At this point it becomes evident once again, that the task of securing the digital heritage concerns an interdisciplinary level.

Central in the Charter are furthermore article 4 and 5. While the former article highlights the importance of immediate action by the member states, in order to avoid "the loss of the digital heritage" which "will be rapid and inevitable", Article 5 underlines the importance of longevity and hence the dominant goal of creating a sustainable, digital memory. The article reads as follows:

“Continuity of the digital heritage is fundamental. To preserve digital heritage, measures will need to be taken throughout the digital information life cycle, from creation to access. Long-term preservation of digital heritage begins with the design of reliable systems and procedures which will produce authentic and stable digital objects”.

Here it becomes clear, that in some way every article of the Charter stands as a concept for itself and at the same time contributes to the greater goal of creating an accessible institutionalized digital memory. While this statement in the fifth article remains relatively abstract, the importance of the lifecycle of the digital heritage becomes evident. Hence, the law in a first step not only must retrace and understand the production of the content, but also must represent and protect the further steps of distribution, modification and storage. Within this framework, however, the interaction of different areas of law is necessary to reflect the diversity of practices, content and technological conditions [42].

In the context of these brief observations on the content of the Charter, it becomes clear that in addition to the content itself, the UNESCO poses questions about the role of sustainable technology in particular. At this stage, however, the text does not provide answers, but leads to further ambiguities.

Finally, and besides the perspective of international law, copyright law may well play a major role in the advancement of the preservation of digital cultural heritage. Whilst the technology is advancing the possibilities to a great extent and in an unprecedented pace, a national based copyright law is often limiting the scope and use [43, p. 343] of the works enough. Not to forget is the fact, that in some regions, such as the European Union, all digital preservation activities take place within a ‘complex and often contradictory legislative landscape’ [44, p. 18] topped by international treaties and obligations.

Although this can be seen as a disadvantage, it is nevertheless also an advantage, as it enables open-ended negotiations on the constitution of a digital memory. Starting from theoretical considerations, the discussion can then be further pursued on a practical level.

## Digital Cultural Heritage as Constitutive Element of Memory?

Hence, the first step in the following is to develop theoretical principles of "memory", which can be used to gain a better understanding of the complexity a possible "digital cultural memory" embodies. Memory in its social, collective and cultural forms as well as its significance for the identity of society have been extensively studied in the literature, where it stands out that culture is intrinsically connected to memory [45, p. 97]. The 2020 global pandemic reminds us of the importance of maintaining and accessing digital cultural memory not only at present but also for future generation. At the same time, we have to acknowledge, that the preservation of the digital heritage—closely linked to digital cultural memory—has been just as lacking in 2020 as in the past decades. Nevertheless, it is only hesitantly addressed by the scientific community. As Astrid Erll [46] notes, there are three main components that underline the relevance of memory theory in current discussions: Firstly, the historical transformation processes play a significant role, especially concerning the memory of witnesses of historical events. Secondly, there is great importance for the scope of the history of science to expand from cultural studies into the individual disciplines in the humanities. Thirdly and most importantly, media technology is ever-evolving; its effects include various new possibilities of storage, it is complex and has the ability to form a collective memory [46, pp. 3–4]. In the following paragraph, we will introduce some concepts essential in research on memory and culture in central Europe.

Next to Aleida and Jan Assmann, Pierre Nora is of particular importance in this context. In his seven-volume major work [47] he developed his theoretical conception of memory. The author argues, that a natural collective memory does not exist anymore. Instead, national memories create collective identity and function as a placeholder for the no longer existing collective memory [46, p. 20]. Central to this theory are the *lieux de mémoire*, which take on the role of memory [48]. These *lieux de mémoire* include not only locations, but also time periods and all objects that evoke memories. They are defined by three inherent and essential dimensions: the material, the functional, and the symbolic dimension. Following this theory, a material object, period, or location that fulfills a social function and embodies a symbolic meaning can be classified as *lieu de mémoire* [46, p. 21]*.*

But what would define a *lieu de mémoire* of the digital age? Would it manifest itself in a server farm or data cloud? Next to a material data carrier, the society—preserving the digital culture of the twenty-first century—must play its corresponding counterpart to add the functional and symbolic dimension. Adding on, traditional cultural institutions, mirroring a part of society, play a crucial role in forming the *lieux de mémoire* of the digital age. At the same time, by incorporating digitization projects into their scope of work, cultural institutions currently are struggling to maintain access to the cultural memory they have collected and preserved on the one hand, and are battling to explore the ever-growing amount of unseen *dark data* [49] on remote servers on the other hand. This ever-growing amount of data and digitized cultural heritage can also fit to another aspect Nora highlights in his later writing, the “explosion patrimoniale”—here he emphasizes the quality of heritage objects to infinitely multiply [50]. Choosing the type of media for safeguarding digital cultural memory is coupled to compromise. Finding the right balance between a potential flood of information and the loss of digital content is an inherent problem of researching digital cultural memory. Here, the question arises whether memories in digital records should be perceived as non-human and hence asocial [51, p. 41]. Due to the fact that a cultural memory can evidently be formed within digital records, a certain social function cannot be denied. Here, a differentiation between a memory in motion and a static technological memory could be considered helpful for further studies. Whereas memory in motion could be an ongoing task for the *information management society* [52, p. 2] as a whole, static memory could be stored in archives in order to be ‘preserve[d] […] for an indefinite time’ [53, p. 93].

We subsequently focus on the work of Aleida and Jan Assmann, who have further shaped the discussion about memory institutions primarily in central Europe and further developed the most extensive concept in international contexts [46, p. 11]. In one of his major works [54], Jan Assmann elaborates on the idea of collective memory. Similar to the components of relevance for memory theory by Erll earlier mentioned, there are three aspects for Aleida and Jan Assmann that in reference to the past, substantiate societal collective and thus cultural memory: Scientific and historical research, media-supported memory, and passing on historical information through contemporary witnesses [54, p. 56] [55, pp. 19–23] [46, p. 3]. Jan Assmann dissects the idea of collective memory into communicative memory and cultural memory [56]. While communicative memory is based on oral traditions, cultural memory comprises the archaeological and written heritage [54, p. 48ff]. The purpose of the latter is to make an unbiased contribution to preserve documents that would otherwise disappear [57, p. 10f]. It thus complements communicative memory, which can fulfil its task only over a period of 3–4 generations, covering about 80–100 years. As Assmann himself states, ‘Cultural memory is a kind of institution. It is exteriorized, objectified, and stored away in symbolic forms that, unlike the sounds of words or the sight of gestures, are stable and situation-transcendent: They may be transferred from one situation to another and transmitted from one generation to another’ [58, p. 111].

Hence, according to Jan and Aleida Assmann, our memories are culturally ‘embedded’ [59, p. 21]. In this way they emphasize that not only social interactions between individuals, but also interactions with objects can produce memories. For individuals, these objects could be images or places. Society however, tends to transfer this role to e.g. monuments and institutions. During this process, any individual may contribute to communicative memory, however contributions to the cultural memory are usually informed ones. The latter involve a scientific interpretation of the past in light of the present and thus fulfill social functions [55, p. 137]. According to Jan Assmann, [60] cultural memory therefore consists of six essential characteristics: The concretion of identity, its capacity to reconstruct, as well as formation, organization, obligation, and reflexivity [60, pp. 129–132]. The concretion of identity means that a group can recognize/derive/identify their identity given the cultural memory. Cultural memory has a capacity to reconstruct, as ‘it always relates its knowledge to an actual and contemporary situation’ [60, p. 130]. Formation separates cultural memory from communicative memory and is achieved by objectivating the meaning. Organization denotes the institutionalization of cultural memory and the cultivation, ‘the specialization of the bearers of cultural memory’ [60, p. 131]. Obligation results in a structure consisting of ‘a clear *system of value* and *differentiations of importance*’ [60, p. 131]. Finally, cultural memory is reflexive of common practice, of the group’s self-image, and it is self-reflexive [60, p. 131]. Aleida Assmann later extended Jan Assmann’s theory by including texts, images, and forgotten documents in archives [55, p. 130ff]. In addition to the expansion, Aleida Assmann splitted cultural memory into functional and storage memory. The first conscious form of memory comprises meaningful elements that can be coherently connected and understood. In this way it makes experiences accessible by sensibly assembling them [55, p. 134]. In contrast, storage memory merely holds together disjointed and amorphous elements. They can however be processed and interpreted to become accessible and connected to functional memory. Once they are composed, constructed, and connected, they gain a meaning (which they formerly lacked in storage memory) and enter functional memory in this new form [55, pp. 134–137].

In this context, digital media acts as a subtype of storage memory that enables society to access it and expand its functional memory. Digitization combined with Assmann’s three criteria of composure, construction, and connection [55, p. 137] therefore opens the door for memory institutions to stronger engage with a transition from memory formerly in the storage memory to functional memory.

## A Rise of a Digital Cultural memory?

Approaching the digital cultural memory has two objectives. The current century must not become an undocumented period without a trace [61, p. 75]. Yet, it is also clear that both the storage of all data, as well the loss of all data, seem to be two unlikely scenarios [62, p. 7]. At the same time, with files being automatically deleted from servers, the continuous danger of losing content is more real than some might expect. We need to enable a conscious transfer of memory from the storage memory to the functional memory to ultimately make the expanding digital cultural memory accessible in the digital age and maintain its structure.

As Aleida Assmann appropriately states, ‘cultures create a contract between the living, the dead, and the not yet living’ [45, p. 97]. In this way, she underlines the role of mankind to actively contribute to the process of archiving and, more far-reaching, the production of meaning. Considering digital cultural heritage, one identifies the role of human being when developing, producing and storing it, but also its role in the establishment of a renewed comprehension of heritage. As such, digital cultural memory not only consists of data in archives, but also of everyday-practices [63, p. 128] and is as such volatile: The digital world is per se short-lived. Trends, images, and opinions change as quickly as ever, data are erased and the overflow of information may create ambiguity, either in memory institutions. The twenty-first century shapes digital cultural heritage and yet the concept of *Cultural Memory* may assume stable and united knowledge of the society’s reflection.

What also becomes clear is that we will not be able to transmit every bit and byte of our digital life. Like other remains which rot or decay, parts of digital cultural heritage get lost or will not be accessible anymore, this loss is sometimes seen as a ‘betrayal’ [64, p. 11f]. Digital cultural heritage as a combination of hard- and software as well as contents and practices face further threats as organic and non-organic remains: In order to experience the content, software, hardware and a medium—such as a screen—are needed. These matters of fact on the one hand and the awareness of cultural heritage in all its forms on the other one raise questions the mankind can’t answer looking at former experiences. What should be remembered [65, p. 441ff] and on what ground do we build these decisions? In light of these prerequisites, must we start to think about ways to forget [66, p. 181ff]? Or ways of more comprehensive curation [67, p. 99ff]? And what about accidents and breakdowns: for example, are computer viruses a part of the digital culture [68]?

These questions not only concern museums, libraries or archives, but society as a whole. It is clear that the decisions to be made will be hard ones, especially as they challenge our notions of memory, society or authorship. Furthermore, it is also clear, that once these decisions will be made, the aim to implement said strategies will add further difficulties.

Nevertheless, optimism is indicated: the digitalization of culture and society offers a great opportunity of creating strong and inspiring bonds between society, its institutions and companies [69, p. xiii] involved. If strategies that are easy-to-follow, clear and binding will be implemented, a sustainable relationship between the actors and a long-term safeguarding of the digital culture can be the result.

## References

[1] Paul C (2011). Digital Art.

[2] Allen-Robertson J (2017). The materiality of digital media: The hard disk drive, phonograph, magnetic tape and optical media in technical close-up. New Media & Society.

[3] Kwet M (1976). Communication and Cultural Domination.

[4] Kwet M (2019). Digital colonialism: US empire and the new imperialism in the Global South. Race & Class.

[5] Goldsmith J, Wu T (2006). Who Controls the Internet?: Illusions of a Borderless Worldn.

[6] L. Monti, G. Delnovo, S. Mirri, P. Salomoni and F. Callegati (2018). Digital Invasions Within Cultural Heritage: Social Media and Crowdsourcing. In Guidi, B., Ricci, L., Calafate, R., Gaggi, O., Marquez-Barja, J. (Eds)., *Smart Objects and Technologiesfor Social Good* (pp. 102–111). Cham: Springer

[7] Clifford J (1997). Routes: travel and translation in the late twentieth century.

[8] Gere C (1997). Museums Contact Zone and the Internet.

[9] Cloonan MV (2015). Frameworks fo Digital Preservation in Preserving our heritage. Perspectives from antiquity to the digital age.

[10] Foster A, Rafferty P (2016). Managing Digital Cultural Objects. Analysis, Discovery And Retrieval.

[11] Carreras C, Mancini F (2014). A story of great expectations: past and present of online/virtual exhibitions. DESIDOC Journal of Library and Information Technology.

[12] Warren E, Matthews G (2019). Public libraries, museums and physical convergence: Context, issues, opportunities: A literature review Part 1. Journal of Librarianship and Information Science.

[13] Evens T, Hauttekette L (2011). Challenges of digital preservation for cultural heritage institutions. Journal of Librarianship and Information Science.

[14] D. DeGroot 2005. "Keeping Our People Alive: The Role of Digital Immortality in Culture Preservation" In T. Dreier, and E. Euler (eds.),Kulturelles Gedächtnis im 21. Jahrhundert: Tagungsband des internationalen Symposiums: 23. April 2005 Karlsruhe, Karlsruhe, Universitätsverlag Karlsruhe.

[15] J. Ekholm and K. Weckström, "Popularity of museums rising all over the world" 13 Dec 2016. [Online]. Available: https://www.stat.fi/uutinen/popularity-of-museums-rising-all-over-the-world. Accessed 20 July 2020.

[16] Saaze V, Wharton G, Reisman L (2018). Adaptive institutional change: Managing digital works at the museum of modern art. Musuem and Society.

[17] Kim J (2020). The archive with a virtual museum: The (im)possibility of the digital archive in Chris Marker's Ouvroir. Memory Studies.

[18] Cane S, Richmond Alison, Bracker Alison (2009). Why do we conserve? Developing an Understanding of Conservation as a Cultural Construct. Conservation: Principles, Dilemmas and Uncomfortable Truths.

[19] Respini E (2018). Art in the Age of the Internet.

[20] P. Laurenson, "Authenticity, Change and Loss in the Conservation of Time-Based Media Installations" Tate Papers no. 6, London, Autumn 2006.

[21] Library of Congress, "Update on the Twitter Archive at the Library of Congress" 12 2017. [Online]. Available: https://blogs.loc.gov/loc/files/2017/12/2017dec_twitter_white-paper.pdf. [Accessed 23 07 2020].

[22] B. Ciecko, 4 Ways Museums Can Successfully Leverage Digital Content and Channels during Coronavirus (COVID-19) 25 03 2020. [Online]. Available: https://www.aam-us.org/2020/03/25/4-ways-museums-can-successfully-leverage-digital-content-and-channels-during-coronavirus-covid-19/. [Accessed 24 07 2020].

[23] Network of European Museum Organisations, "Map of digital responses by museums during the Coronavirus pandemic" 22 07 2020. [Online]. Available: https://www.ne-mo.org/news/article/nemo/map-of-digital-responses-by-museums-during-the-coronavirus-pandemic.html. [Accessed 24 07 2020].

[24] I. Grevtsova, "10 Insights about the future of technology during the post-COVID-19 era. Interview with Conxa Rodà" 30 06 2020. [Online]. Available: https://irinagrevtsova.com/en/10-insights-sobre-el-futuro-de-las-nuevas-tecnologias-en-la-era-post-covid-19-entrevista-a-conxa-roda/. [Accessed 24 07 2020].

[25] Paloques-Berges P, Schafer V (2015). Quand la communication devient patrimoine. Hermès, La Revue.

[26] De Kosnik A (2016). Rogue Archives: Digital Cultural Memory and Media Fandom.

[27] Clough G (2013). Best of Both Worlds: Museums, Libraries, and Archives in a Digital Age.

[28] F. R. Cameron 2020 Theorising heritage collection digitisations in global computational infrastructures" in The Routledge International Handbook of New Digital Practices in Galleries, Libraries, Archives, Museums and Heritage Sites, London / New York, eds. Lewi H., Smith W., vom Lehm D. and Cooke S. Routledge, pp. 55–67.

[29] M. Lee, “360° Mobile VR Research Projects for Interactive Media Art Installations”, [Online]. Available: https://marclee.io/en/vr-mobile-apps/. [Accessed 24 Juli 2020].

[30] A.-K. Riedl and M. Hunstig, "Museumsrundgang mit der Chefredakteurin: Christiane Arp führt Sie exklusiv durch die virtuelle VOGUE-Ausstellung" 15 Mai 2020. [Online]. Available: https://www.vogue.de/lifestyle/artikel/40-jahre-vogue-ausstellung-museumsrundgang-digital-museum?fbclid=IwAR24r2P22ljQ3OpTUcnD8ji76m1nPhYwWNz0Cnog8mP8LZ-vYkRLiH4dnE8. [Accessed 24 Juli 2020].

[31] M. Buhrs and A. Marr, "Die demokratische Schnecke. Ein großes Märchen" 22 April 2020. [Online]. Available: https://www.diedemokratischeschnecke.de. [Accessed 24 Juli 2020].

[32] Foundation F (2020). The Aura in the Age of Digital Materiality Rethinking Preservation in the Shadow of an Uncertain Future.

[33] Compare here the “Metall auf Metall” decision, Bundesverfassungsgericht (Federal Constitutional Court), Judgment of the First Senate of 31 May 2016 – 1 BvR 1585/13 – paras. 1-125, BVerfGE 142, 74.

[34] de Lusenet Y (2007). Tending the Garden or Harvesting the fields: Digital Preservation and the UNESCO charter on the preservation of the digital HERITAGE. Library Trends.

[35] Operational Directives for the Implementation of the Convention for the Safeguarding of the Intangible Cultural Heritage. Adopted by the General Assembly of the States Parties to the Convention at its second session (UNESCO Headquarters, Paris, 16 to 19 June 2008), amended at its third session (UNESCO Headquarters, Paris, 22 to 24 June 2010), its fourth session (UNESCO Headquarters, Paris, 4 to 8 June 2012), its fifth session (UNESCO Headquarters, Paris, 2 to 4 June 2014), its sixth session (UNESCO Headquarters, Paris, 30 May to 1 June 2016) and its seventh session (UNESCO Headquarters, Paris, 4 to 6 June 2018), version 7.GA (2018).

[36] Stainforth E, Carter T (2019). Disruptive forms, persistent values: negotiating digital heritage and the memory of the world. Creating Heritage: Unrecognised Pasts and Rejected Futures.

[37] Prodan AC, Edmondson R, Jordan L, Prodan AC (2019). Memory of the World, Documentary Heritage and Digital Technology: Critical Perspectives. The UNESCO Memory of the World Programme: Key Aspects and Recent Developments.

[38] UNESCO, "Recommandation concerning the preservation of, and access to, documentary heritage including in digital form" General Conference, 38th session, 3–8.11.2015, 38 C/Resolution 55, 2015.

[39] [Online]. Available: https://www.softwareheritage.org/. [Accessed 21 Juli 2020].

[40] Shyllon F (2012). Expert Meeting on the 20th Anniversary of UNESCO's Memory of the World Programme: Warsaw, Poland, 8–10 May 2012. International Journal of Cultural Property.

[41] Maget Dominicé A, Haux DH, Graf FS (2020). Saving Content in Digital Surroundings: A Safe Solution?. Pólemos.

[42] R. H. Weber and L. Chrobak, "Legal Implications of Digital Heritagization" RESET. Recherches en sciences sociales sur Internet, vol. 6, 2017. 10.4000/reset.826.

[43] Euler E (2011). Das kulturelle Gedächtnis im Zeitalter digitaler und vernetzter Medien und sein Recht: Status Quo der rechtlichen, insbesondere urheberrechtlichen Rahmenbedingungen von Bestandsaufbau, Bestandserhaltung und kommunikativer sowie kommerzieller Bestandsverm.

[44] Anderson D (2013). Preserving europe's digital cultural heritage: A legal perspective. New Review of Information Networking.

[45] Assmann A, Erll A, Nünning A (2010). Canon and Archive. A Companion to Cultural Memory Studies.

[46] Erll A (2017). "Kollektives Gedächtnis und Erinnerungkulturen" Stuttgart.

[47] Nora P (1984). Les lieux de mémoire.

[48] Nora P (1984). Les lieux de mémoire: 1 La République.

[49] Schembera B, Durán J (2020). Dark data as the new challenge for big data science and the introduction of the scientific data officer. Philosophy & Technology.

[50] Nora P, Sire M-A (1996). Préface. La France du patrimoine, Les choix de la mémoire.

[51] Ernst W, Blom I, Lundemo T, Røssaak E (2016). Electrified Voices: Non-Human Agencies of Socio-Cultural Memory. Memory in motion: archives, technology, and the social.

[52] Parikka J, Ernst W, Parikka J (2012). An Introduction to Wolfgang Ernst’s Media Archaeology. Digital Memory and the Archive.

[53] Ernst W, Kyong Chun WH, Keenan Thomas (2016). Dis/continuities: Does the Archive Become Metaphorical in Multi-Media Space?. New Media Old Media: A History and Theory Reader.

[54] Assmann J (1992). Das kulturelle Gedächtnis: Schrift, Erinnerung und politische Identität in frühen Hochkulturen.

[55] Assmann A (1999). Erinnerungsräume Formen und Wandlungen des kulturellen Gedächtnisses.

[56] Assmann J, Assmann J, Hölscher T (1988). Kollektives Gedächtnis und kulturelle Identität. Kultur und Gedächtnis.

[57] Pfleiderer M, Pfleiderer M (2011). Populäre Musik und kulturelles Gedächtnis. Zur Einführung. Populäre Musik und kulturelles Gedächtnis. Geschichtsschreibung—Archiv—Internet Köln.

[58] Assmann J, Erll A, Nünning A (2008). Communicative and Cultural Memory. Media and Cultural Memory.

[59] J. Assmann, Der Begrif des kulturellen Gedächtnisses in Kulturelles Gedächtnis im 21. Jahrhundert: Tagungsband des internationalen Symposiums: 23. April 2005, Karlsruhe, eds. T. Dreier and E. Euler, Karlsruhe: Universitätsverlag Karlsruhe, 2005, pp. 21–29.

[60] J. Assmann, “Collective Memory and Cultural Identity” New German Critique , Spring–Summer, 1995, No. 65, Cultural History/Cultural Studies (Spring—Summer, 1995), pp. 125–133, pp. Translation of J. Assmann, „Kollektives Gedächtnis und kulturelle Identität“ in Kultur und Gedächtnis, Frankfurt a.M., eds. J. Assmann, T. Hölscher. Suhrkamp, 1988, pp. 9–19.

[61] T. Dreier, Informationsrecht in der Informationsgesellschaft. In *Umbruch von Regelungssystemen in der Informationsgesellschaft: Freundesgabe für Alfred Büllesbach*, eds. J. Bizer, B. Lutterbeck, and J. Rieß, Stuttgart: J.F. Steinkopf, pp. 65–76.

[62] T. Dreier, "Kulturelles Gedächtnis—Digitales Gedächtnis—Eine Einführung" In Kulturelles Gedächtnis im 21. Jahrhundert: Tagungsband des internationalen Symposiums: 23. April 2005 Karlsruhe, Karlsruhe. T. Dreier, E. Euler, (eds.), Universitätsverlag Karlsruhe, 2005.

[63] M. Heck, "Städtische Kulturpolitik und kulturelles Gedächtnis" in Kulturelles Gedächtnis im 21. Jahrhundert: Tagungsband des internationalen Symposiums: 23. April 2005, Karlsruhe, Karlsruhe, T. Dreier, E. Euler (eds.), Universitätsverlag Karlsruhe, 2005.

[64] Blom I, Blom I, Lundemo T, Røssaak E (2017). Rethinking Social Memory: Archives Technology and the Social. Memory in Motion: Archives Technology and the Social.

[65] Ashuri T (2011). (Web)sites of memory and the rise of moral mnemonic agents. New media & society.

[66] Esposito E, Erll A, Nünning A (2008). Social Forgetting: a systems theory approach. Media and Cultural Memory.

[67] S. Ross, "The Challenges of Digital Curation and Approaches to Meeting Them: The Role of ERPANET" in Kulturelles Gedächtnis im 21. Jahrhundert: Tagungsband des internationalen Symposiums: 23. April 2005, Karlsruhe, Karlsruhe, eds. T. Dreier, E. Euler. Universitätsverlag Karlsruhe, 2005.

[68] Parikka J (2007). Digital Contagions: A Media Archaelogy of Computer Viruses.

[69] Council of Canadian Academies (2015). Leading in the Digital World: Opportunities for Canada’s Memory Institutions Ottawa ON, The Expert Panel on Memory Institutions and the Digital Revolution.

